# Editorial: 365 days of progress in neuro-oncology and neurosurgical oncology

**DOI:** 10.3389/fonc.2023.1279957

**Published:** 2023-09-07

**Authors:** Erik P. Sulman, David D. Eisenstat

**Affiliations:** ^1^ Section of Neuro-oncology & Neurosurgical Oncology, Frontiers in Oncology and Frontiers in Neurology, Lausanne, Switzerland; ^2^ Department of Radiation Oncology, NYU Grossman School of Medicine, New York, NY, United States; ^3^ Brain and Spine Tumor Center, Laura and Isaac Perlmutter Cancer Center, New York, NY, United States; ^4^ NYU Langone Health, New York, NY, United States; ^5^ Children’s Cancer Centre, Royal Children’s Hospital, Parkville VIC, Australia; ^6^ Stem Cell Biology, Murdoch Children’s Research Institute, Parkville, VIC, Australia; ^7^ Department of Paediatrics, University of Melbourne, Parkville, VIC, Australia

**Keywords:** brain tumor, prognostic factor, biomarker, palliative care, tumor treating fields

The Frontiers Research Topic titled *365 Days of Progress in Neuro-Oncology and Neurosurgical Oncology* published a collection of ten articles from August 2022 to March 2023. The topics contributed to the field of Neuro-Oncology on a variety of subjects focused on primary central nervous system (CNS) tumors as well as brain metastases, from tumor treating fields, palliative care for glioma patients and evaluation of clinical and/or biomarkers for risk or prognostic assessment for several CNS tumor entities.

## Clinical prognostic factors for less common central nervous system tumors

The Surveillance, Epidemiology, and End Results (SEER) Program, funded by the USA-based National Cancer Institute (NCI), is a major cancer statistics database. Three separate analyses of the SEER population-based databases were submitted to this Research Topic. Zhang Z. et al. reviewed the SEER database for the relatively uncommon entity, *intracranial subependymoma*, from 2004-2016, and established a prognostic nomogram. Of 667 evaluable patients, 535 patients were assigned to the training cohort and 132 into the validation cohort. Of interest, only age and gender were independent prognostic factors for overall survival (OS); extent of resection, tumor location, grade, size and radiation treatment were not significant. Potential limitations of the study included exclusion of patients with acute deaths (i.e. survived less than a month), patient selection bias, and a median follow-up of 56 months. Validation in an independent cohort will be necessary. Zhang Z. et al. studied 413 patients with *central neurocytoma*, using the SEER database from 2003-2019, with specific attention to tumor size, the extent of resection and/or adjuvant radiation therapy (RT). The investigators demonstrated better outcomes for patients with smaller tumors (less than 4 cm), gross total resection (GTR) or for those who did not receive RT, especially after a GTR. Outcomes after RT were worse for patients with extraventricular central neurocytoma. Similar to the other study, limitations included exclusion of patients with acute deaths. However, a major concern acknowledged by the authors was the change in diagnostic criteria for central neurocytoma during the study period, including revisions to the World Health Organization (WHO) CNS tumour classification in 2007, 2016 and most recently in 2021 (1). Validation in an independent clinical patient cohort with central neuropathology review using the WHO 2021 criteria are important next steps.

Liu et al retrospectively evaluated 18 cases of *secondary gliosarcoma* from their institution, diagnosed from 2013 to 2020 in patients with pre-existing gliomas. The authors also included 89 cases from 39 publications from the existing literature and applied PRISMA guidelines (2). As expected, patients who were less than 60 years or with a non-GBM initial diagnosis had longer periods of disease progression to secondary gliosarcoma. Ten of 107 patients had extracranial metastases (9.4%); the lungs were the most common site. Better outcomes were experienced by secondary gliosarcoma patients with a GTR and adjuvant chemoradiation. Study limitations also include changes in the diagnostic criteria for gliosarcoma for the institutional cases and those identified in the included published series.

## Impact of patient and treatment-related factors on patient outcomes

Jin et al. also utilized the SEER database to evaluate factors contributing to cerebrovascular mortality in 72,916 patients diagnosed with a glioma from 2000 to 2018. The investigators applied the STROBE (Strengthening the Reporting of Observational Studies in Epidemiology) guidelines (3).

In this retrospective, observational cohort study, surgery and chemotherapy had significantly decreased, whereas higher tumor grade (Grade 4 versus Grade 2) and larger tumors (greater than 3 cm) had significantly increased cerebrovascular mortality. Of particular interest for the field of CNS tumor survivorship was the association of radiotherapy with a higher risk of cerebrovascular mortality in those surviving 5 or more years from their cancer diagnosis. Study limitations include probable under-reporting of cerebrovascular events in patients with a glioma as well as the challenges of making a diagnosis of stroke in brain tumor patients, both clinically and neuroradiologically. Furthermore, the SEER database does not provide baseline cardiovascular risk factors which may impact both the incidence and outcomes of older patients with gliomas. However, this study identifies multiple avenues for future research.

Two reports examined patients with metastases to the brain and spine, respectively. Yu et al retrospectively studied the impact of smoking on the prognosis of 2,647 lung cancer patients with brain metastases from 2013 to 2021. Surprisingly, 67.1% declared no smoking history but this data was extracted from the electronic medical record and was not validated by patient self-report or by other means. Current and former smokers had an increased risk of death when compared to never smokers. Furthermore, quitting smoking did not correlate with better survival outcomes in this lung cancer patient cohort with established brain metastases. However, the authors suggest that increasing the accumulated smoking cessation time prior to a diagnosis of brain metastasis may improve patient survival. Validation of this study in other patient cohorts and from other countries is required. Hamed et al. retrospectively evaluated postoperative interventions and 30-day and 1 year mortality outcomes in 198 patients with spinal metastases treated surgically treated from 2015-2019 at a single institution. Postoperative mechanical ventilation (PMV) was considered prolonged if its duration was greater than 24 hours. Twenty patients (10%) had prolonged PMV; they experienced 70% and 100% mortality at 30-days and 1-year, respectively. PMV greater than 24 hr was the sole independent predictor for 30-days mortality. Given that the patient cohort with prolonged PMV was small, this report warrants further study in multiple centres.

## Utility of clinical algorithms or biomarker-based signatures to predict patient outcomes

Biomarker discovery has been enhanced by the wider availability of RNA sequencing data that include microRNAs and long noncoding RNAs (lncRNAs), operationally defined as longer than 200 nt. Using the TCGA and GTEx databases, Song et al. focused their study on necroptosis-related lncRNAs in a cohort of patients with IDH-wild-type glioblastoma (GBM). The authors identified six necroptosis-related lncRNAs and then generated a prognostic lncRNA signature as well as investigated the associated immune-related tumor microenvironment. One lncRNA, RP11-131L12.4, was inversely correlated with OS in patients and the level of necroptosis *in vitro*. The authors suggest that targeting necroptosis-related ncRNAs may be a useful adjunct to current immunotherapy approaches under investigation for IDH-wild-type GBM.

El-Hajj et al. sought to assess the utility of the MAC-score to preoperatively predict an increased MIB-1 index (greater than 5%) in 108 spinal menigioma patients. The MIB-1 index is a semi-quantitative measurement of immunolabeling of Ki-67 of formalin-fixed paraffin embedded (FFPE) tissue sections. The MAC-score for spinal meningioma adds to the modified McCormick (mMC) scale that is in wide clinical use by adding 1 point for a preoperative mMC ≥ 2 (M), 1 point for age ≥ 65 years (A), and 2 points for the absence of calcification within the tumor (C). The authors were unable to externally validate the MAC-score and discuss potential methodological issues with the study by Wach et al. (4).

## Real world experience with tumor-treating fields

Tumor-Treating Fields (TTFields) are an FDA-approved treatment for newly diagnosed and recurrent GBM. However, evaluation of their use in many countries is ongoing. She et al retrospectively evaluated 52 newly diagnosed and 41 recurrent GBM patients from a single-center in China; 13 patients in each group received TTFields. The authors concluded that TTFields provided a clinical benefit in newly diagnosed but not in recurrent GBM, especially in patients who had a subtotal resection (STR). However, this is a small single-institution study and the authors advocate for further multi-institutional studies.

## Bibliometric assessment of the role of palliative care in patients with glioma

Recently, there have been additions to meta-analyses and systematic reviews, including bibliometric analyses of publication databases, to provide a summary of current research and identify areas for future study. Xiao et al. used a type of bibliometry, known as scientometric analysis, on the topic of palliative care for glioma patients. The authors applied the PRISMA guidelines to this topic using the Web of Science database for the year of 2022 and selected the top-100 most cited papers from 2,542 articles. They identified a variety of palliative care needs for glioma patients as well as their caregivers. Furthermore, they observed few randomized controlled trials in palliative care in this patient group.

## Concluding remarks

The selected topics included in this Research Topic in Neuro-Oncology and Neurosurgical Oncology provide a selected snapshot of current clinical and translational research activities. Of significance, many of these studies seek to identify clinical variables and/or biomarkers in various CNS tumor classes. Unfortunately, the established large databases have not incorporated revised molecular genetic criteria to confirm the neuropathological diagnostic entities listed in the updated 2021 WHO CNS tumor classification (1). Hence, some of the conclusions of the included manuscripts require validation in other patient cohorts whose diagnoses use the revised WHO CNS criteria. Although the use of the same database for both the identification and validation of prognostic factors, biomarkers and to establish nomograms is widely accepted, few if any novel observations from these types of study have been incorporated into clinical trials or the neuro-oncology clinic. Therefore, investigators in neuro-oncology are highly encouraged to validate these types of studies in another large patient database, confirm identified biomarkers in actual clinical samples and functionally validate them in relevant tumor cell line models and/or patient derived orthotopic xenotransplants as appropriate.

## Author contributions

ES: Writing – review & editing. DE: Writing – original draft, Writing – review & editing.
